# Feasibility of resuscitative transesophageal echocardiography at out-of-hospital emergency scenes of cardiac arrest

**DOI:** 10.1038/s41598-023-46684-x

**Published:** 2023-11-16

**Authors:** Mario Krammel, Thomas Hamp, Christina Hafner, Ingrid Magnet, Michael Poppe, Peter Marhofer

**Affiliations:** 1 Emergency Medical Service Vienna, Radetzkystrasse 1, 1030 Vienna, Austria; 2 PULS Austrian Cardiac Arrest Awareness Association, Lichtentaler Gasse 4/1/R03, 1090 Vienna, Austria; 3 Department of Anesthesia, Intensive Care Medicine and Pain Medicine, Medical University of Vienna, Waehringer Guertel 18–20, 1090 Vienna, Austria; 4 Department of Emergency Medicine, Medical University of Vienna, Waehringer Guertel 18–20, 1090 Vienna, Austria

**Keywords:** Cardiovascular diseases, Echocardiography

## Abstract

Guidelines recommend the use of ultrasound in cardiac arrest. Transthoracic echocardiography, has issues with image quality and by increasing hands-off times during resuscitation. We assessed the feasibility of transesophageal echocardiography (TEE), which does not have both problems, at out-of-hospital cardiac arrest (OHCA) emergency scenes. Included were 10 adults with non-traumatic OHCA in Vienna, Austria. An expert in emergency ultrasound was dispatched to the scenes in addition to the resuscitation team. Feasibility was defined as the ability to collect specific items of information by TEE within 10 min. Descriptive statistics were compiled and hands-off times were compared to a historical control group. TEE examinations were feasible in 9 of 10 cases and prompted changes in clinical management in 2 cases (cardiac tamponade: n = 1; right ventricular dilatation: n = 1). Their mean time requirement was 5.1 ± 1.7 (2.8–8.0) min, and image quality was invariably rated as excellent or good during both compressions and pauses. No TEE-related complications, or interferences with activities of advanced life support were observed. The hands-off times during resuscitation were comparable to a historical control group not involving ultrasound (*P* = 0.24). Given these feasibility results, we expect that TEE can be used routinely at OHCA emergency scenes.

## Introduction

Current guidelines recommend that, once advanced life support has been established, ultrasound should be used to detect potentially reversible causes of cardiac arrest^1^. In prehospital settings, the non-invasive technique of transthoracic echocardiography is normally used, given that small and portable ultrasound systems are available for this technique that can readily be accessed at most scenes of emergency.

Despite suggestions that transthoracic echocardiography may be feasible in prehospital settings and may usually offer adequate image quality during cardiac arrest, its limited ultrasound windows do carry a risk of major quality issues^2,3^. Also, the time available to perform transthoracic ultrasound examinations is limited to short moments when chest compressions are paused for rhythm checks, and transthoracic echocardiography has also been suspected to increase hands-off times during cardiopulmonary resuscitation^4–8^. Transesophageal echocardiography (TEE) has the potential to overcome these issues by not interfering with cardiopulmonary resuscitation, allowing continuous visualization of the heart even during hands-on times, and normally offering a quality of imaging superior to transthoracic ultrasound^9–11^. In addition, TEE has been shown to be highly sensitive in detecting reversible causes of cardiac arrest and may improve chest compressions by identifying the optimal surface position for their application^12^. Several study reports are available on the use of TEE to guide cardiopulmonary resuscitation both in the hospital and following out-of-hospital cardiac arrest (OHCA) in the emergency department^13–15^.

A significant proportion of patients who suffer OHCA will die on the emergency scene without being transported to a hospital. Even in situations where transport under cardiopulmonary resuscitation does take place for further diagnostics and treatment in the hospital, considerable time will be lost until potentially reversible causes are finally detected and treated. TEE examinations in prehospital settings might allow reversible causes of OHCA to be detected earlier and, as a consequence, treatment to be improved. TEE can also be used to direct hands positioning during CPR, but this was not within the scope of this study. Hence, the present study was designed as a case series investigating the feasibility of prehospital TEE to detect potentially reversible causes of cardiac arrest at scenes of OHCA emergency.

## Methods

### Design and setting

This single-center, prospective case series was performed by the Emergency Medical Service of Vienna in cooperation with the Medical University of Vienna between December 2021 and March 2022 in Vienna, Austria. Patient enrolment was preceded by registration at ClinicalTrials.gov (NCT05185596, 11/01/2022) and approval by the institutional review board (Ethics Committee of the Medical University of Vienna, Ref. Nr. 1718/2019), which ruled that an upper limit of 10 patients was not to be exceeded. The institutional review board (Ethics Committee of the Medical University of Vienna, Ref. Nr. 1718/2019) waived the need for informed consent, but surviving patients had to be subsequently informed of their inclusion. The study was performed in accordance with the Declaration of Helsinki and the relevant national regulations.

### Patients characteristics

Inclusion in the study was confined to adult patients who suffered cardiac arrest and were treated by advanced life support—including the use of an endotracheal tube to secure the airway—at OHCA emergency scenes. Patients were excluded if they required immediate transport to a hospital under cardiopulmonary resuscitation (i.e. due to pregnancy, traumatic cardiac arrest, persistent ventricular fibrillation, or for extracorporeal membrane oxygenation) or if TEE was contraindicated (i.e. known esophageal stenosis, esophageal tumor, recent upper gastrointestinal surgery).

### Interventions and measurements

The standard management for OHCA within the city of Vienna is to dispatch an advanced-life-support ambulance with two emergency technicians, a doctors' car staffed with an emergency physician and an emergency technician, and a field supervisor (a senior emergency technician) to the scene of the emergency. For the purpose of our investigation, an additional study team was dispatched, which comprised both an emergency technician and an emergency physician trained in TEE. The study team was available from 7:00 am to 7:00 pm on six days during the study period and, on these days, was dispatched to all calls, for presumed adult out of hospital cardiac arrest.

The primary responsibility for treatment rested with the routinely dispatched team, while the study team was in charge of the TEE examination only. However, when they arrived to the scene first, they immediately started treatment and handed over to the routinely dispatched team upon their arrival. TEE was initiated once the required measures of advanced life support, including endotracheal intubation, were in place.

A portable ultrasound unit was used with its proprietary transesophageal transducer (Sonosite Edge II and Sonosite TEExi ; FUJIFILM Sonosite, Amsterdam, Netherlands). With the probe inserted, a mid-esophageal four-chamber, a mid-esophageal three-chamber, and a trans-gastric short-axis view were obtained to collect four items of information as described below (see *Primary outcome* below). A member of the study team measured the time requirement for the TEE examination from the team's arrival and intubation in place until the four items of information had been collected. This information was then communicated to the standard team, who, depending on the situation and using their discretion, decided whether the TEE probe should be removed (n = 3)**,** TEE continued throughout resuscitation (n = 4), or the patient transported to the hospital (n = 2).

Image quality was rated by the TEE operator on the same five-point scale used for grades throughout the Austrian education system, thus being a natural reference for decisions under time pressure (1 = great/excellent; 2 = good; 3 = fair; 4 = poor; 5 = inadequate). These ratings were obtained both during chest compressions and during pauses.

A member of the study team documented any potential complications related to the TEE procedure such as oropharyngeal injury, dental trauma, malposition of the probe, or inadvertent extubation. The field supervisor, an emergency technician in charge of quality assurance, watched over the entire scenery and documented any effects that the conduct of the TEE examination may have had on the emergency team's performance.

### Outcomes

#### Primary outcome

Feasibility of TEE to guide cardiopulmonary resuscitation at out-of-hospital emergency scenes of cardiac arrest was the primary outcome of this study. The criterion of feasibility was considered to be met when the TEE examination could actually be performed and the following items of information collected within 10 min:

- Myocardial activity and aortic valve opening during compression pauses
- Size and function of the left ventricle (rough estimate)
- Size and function of the right ventricle (rough estimate)
- Presence or absence of pericardial effusion or tamponade

#### Secondary outcomes

- Time requirements for the TEE examinations (duration until completion)
- Image quality as rated by the TEE operator during compressions and pauses
- Changes in clinical management decisions as a result of TEE findings
- Complications of TEE examinations and effects on the team's performance
- Interference of TEE examinations with activities of advanced life support
- Effect of TEE examinations on hands-off times during resuscitation

#### Hands-off times versus a historical control group

Hands-off times were evaluated based on the patient impedance records kept in the internal memory of the defibrillators (Lifepak 15; Physio-Control, Redmond, WA, USA). These hands-off data were compared to a historical control group of patients from the Vienna Cardiac Arrest Registry whose management had not involved point-of-care ultrasound. To this end, each patient who could be examined by TEE was matched with two historical controls based on age, gender, body mass index, initial heart rhythm, setting of the emergency, total duration of cardiopulmonary resuscitation, and use of a mechanical chest compression system (LUCAS®). Hands-off times are expressed in absolute terms and as percentages of the total resuscitation times.

### Analysis

No sample size calculation was performed for the purpose of this feasibility study, also because the ethics committee had ruled an upper limit of 10 patients. Statistical software (SPSS Statistics 27.0; IBM, Armonk, NY, USA) was used for descriptive data analysis (mean values, standard deviations, percentages) and for a *t-*test comparing the hands-off times between the study population and the historical control group. Data are presented as mean ± SD (min–max).

### Ethical Approval

Patient enrolment was preceded approval by the institutional review board (Ethics Committee of the Medical University of Vienna, Ref. Nr. 1718/2019).

## Results

**Figure 1:**
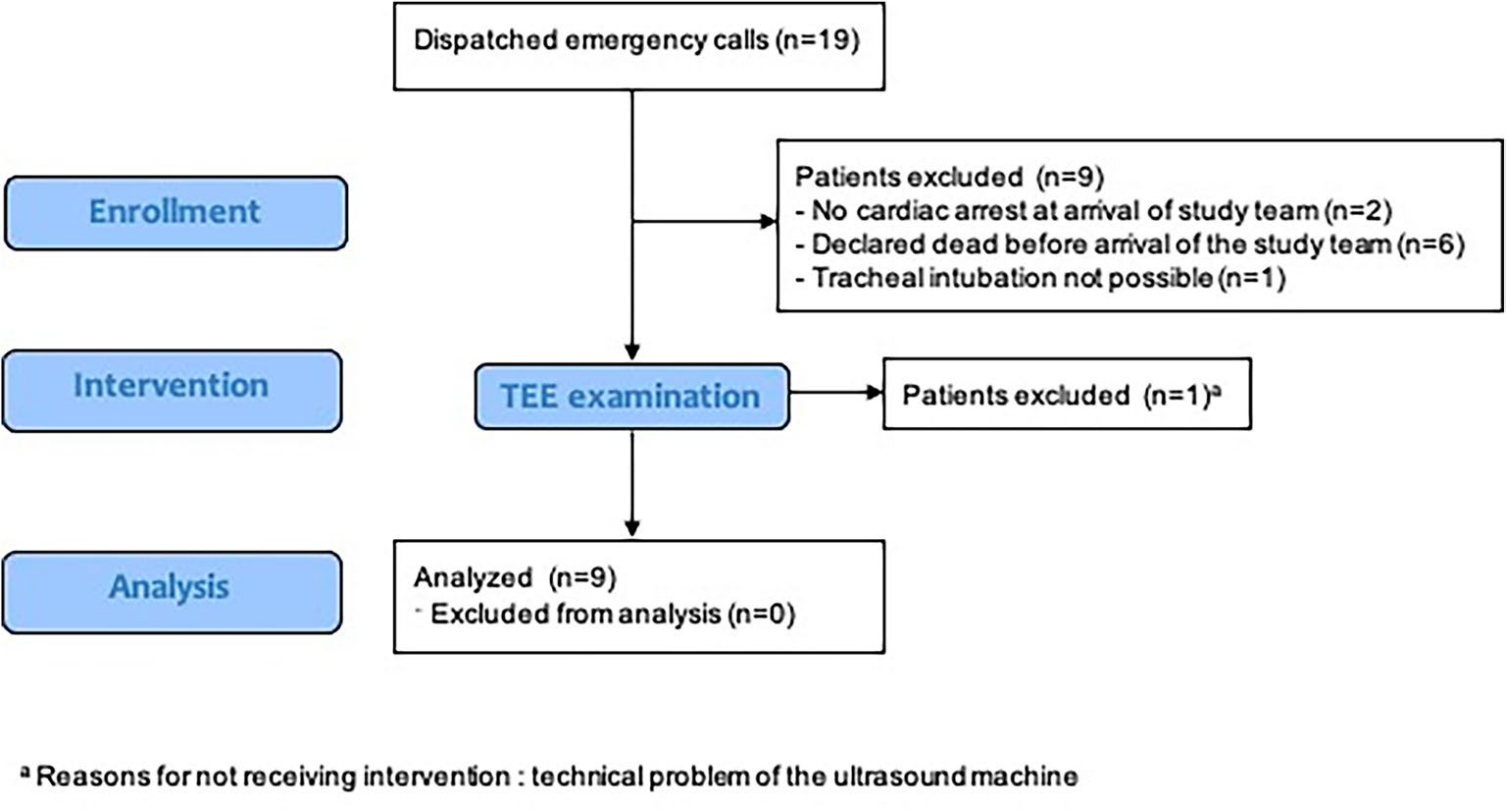
CONSORT diagram of the study design.

The study team was dispatched to 19 emergency scenes of suspected OHCA (Fig. 1). Six of these patients were dead by the time the first team arrived, so that cardiopulmonary resuscitation attempts were stopped without a need to perform a TEE examination. In two patients, resuscitation was not initiated because the suspicion of cardiac arrest was not confirmed. One patient required a supraglottic airway device and could not be included.

Table 1 gives an overview of pertinent patient data for the 10 cases that eventually were included as the upper limit set by the ethics committee. Nine of these met the feasibility criterion that the TEE examination could be completed and the required items of information obtained within 10 min. In one patient, the planned TEE examination was prevented by a power failure of the ultrasound unit. The average time requirement for completion of the TEE examination was 5.1 ± 1.7 (2.8–8.0) min.

**Table 1:** Demographic data, event-related findings, and patient outcomes.

Patient no	1	2	3	4	5	6	7	8	9	10
Age, years	60	70	81	52	56	69	75	75	66	72
Gender	m	m	f	m	m	f	m	f	m	m
BMI	24	22	39	27	37	26	31	25	31	26
Initial ECG rhythm	PEA	PEA	PEA	ASY	PEA	VF	PEA	ASY	ASY	PEA
Presumed origin of cardiac arrest	Card	Resp.	Card	Card	Card	Card	Card	Card	Card	PE
Emergency scene	Home	Public	Home	Home	Business room	Public	Home	Home	Home	Nursing home
On-scene CPR, min	37	42	12	15	28	21	45	10	12	61
Total Hands-off time, s	34	n/a	64	222	107	67	n/a	115	76	231
Fraction of total Hands-off time, %	2	n/a	9	25	6	5	n/a	19	11	6
Hands-off time 5 min after intubation, s	34	n/a	36	40	31	45	16	28	40	8
Fraction of hands-off time 5 min after intubation, %	11	n/a	12	13	10	15	5	9	13	3
Mechanical chest compression system used	Yes	Yes	No	Yes	Yes	No	No	No	No	Yes
Outcome	CPR transport	ROSC	ROSC	Died on scene	CPR transport	Died on scene	Died on scene	Died on scene	Died on scene	ROSC

ASY = asystole; BMI = body-mass index; CPR = cardiopulmonary resuscitation; ECG = electrocardiography; n/a = not applicable; PEA = pulseless electrical activity; ROSC = return of spontaneous circulation; VF = ventricular fibrillation. Card. = presumed cardiac origin, Resp. = presumed respiratory origin, PE = presumed pulmonary embolism.

Average fraction of hands-off time of the total resuscitation time was 10 ± 7 (2–25) % when TEE was performed. This was statistically comparable to a result of 9 ± 5 (2—21) % in the historical control group (P = 0.24) (Table 1).

Average fraction of hands-off time within 5 min after intubation was 10 ± 4 (3–15) % when TEE was performed and 13 ± 3 (6–20) % in the historical control group, without statistical significance (*P* = 0.15) (Table 1).

**Figure 2:**
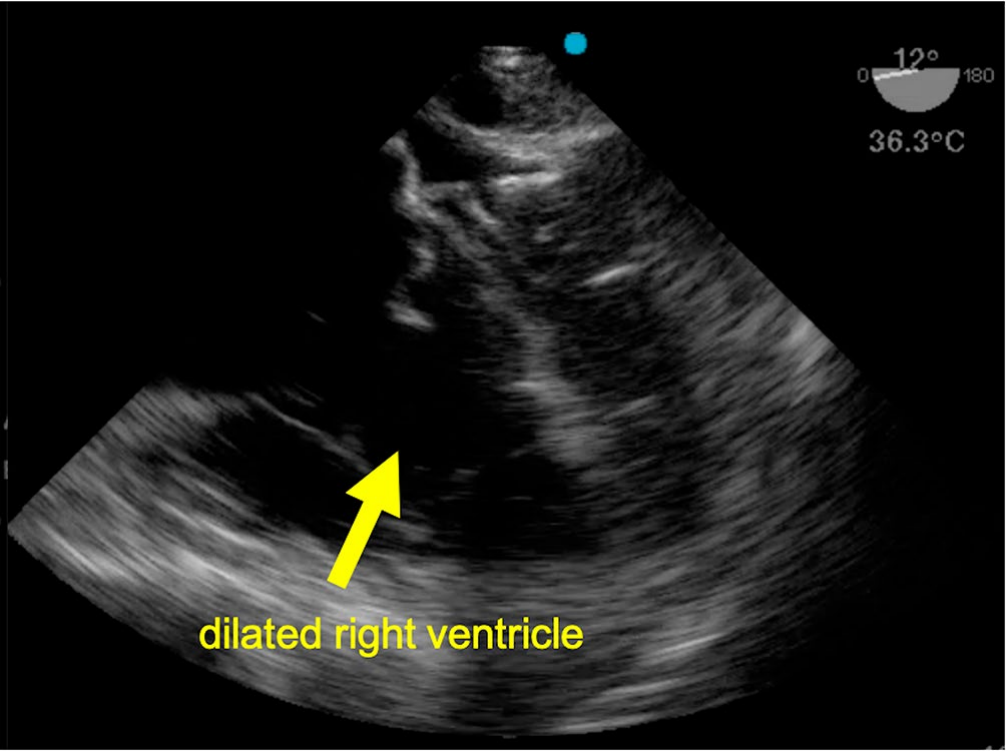
Mid-esophgeal four-chamber view obtained by TEE during cardiopulmonary resuscitation of patient no. 10.

**Figure 3:**
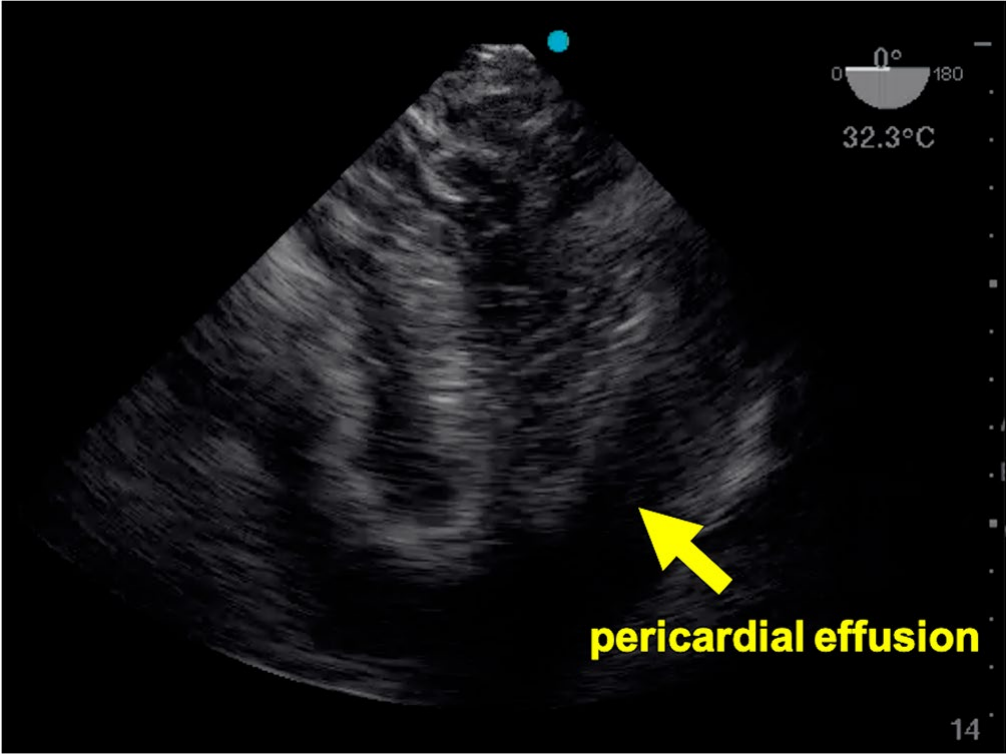
Mid-esophgeal four-chamber view obtained by TEE during cardiopulmonary resuscitation of patient no. 4.

In three cases, the TEE findings prompted changes in clinical management. One return of spontaneous circulation was detected before clinical signs became apparent, resulting in discontinuation of the cardiopulmonary resuscitation before the next rhythm check. The second one of these patients received thrombolysis for suspected pulmonary embolism—with a return of spontaneous circulation 30 min later—after massive dilatation of the right ventricle had been visualized (Fig. 2). The third patient died, despite an attempt at pericardial drainage, after the TEE had revealed pericardial tamponade (Fig. 3).

No complications were noted as a result of the TEE examinations. According to the field supervisor, the standard emergency team's performance was, in one case (the one just mentioned), affected by an unexpected ultrasound finding of massive hemorrhagic pericardial effusion, in that the team had difficulty deciding whether chest compressions should be continued or suspended during the attempted relief of the tamponade. In this case, the fractional hands within the first 5 min after intubation (when the pericardial effusion was detected) was 13% and fractional hands-off time within 10 min after intubation (when the tamponade was attempted to be relieved) was 34%. This suggests, that rather the therapeutic intervention for this unexpected intervention, than the TEE exam itself caused the rather long hands-off time in this case.

Table 2 summarizes the results for the TEE-related outcome parameters. Image quality as rated by the TEE operator was excellent (best out of five possible scores) in six cases and good (second-best out of five possible scores) in three cases during ongoing chest compressions. During pauses, the ratings were likewise excellent in six and good in three cases. In other words, the ratings of image quality did not differ or change between ongoing cardiopulmonary resuscitation and intermittent pauses.

**Table 2:** Findings related to the TEE examinations.

Patient no		1	2	3		4	5	6		7	8			9	10
Feasibility of TEE		Yes	No	Yes		Yes	Yes	Yes		Yes	Yes			Yes	Yes
Duration of TEE, s		238	N/a	250		480	310	231		414	418			165	122
Image quality*															
During CPR		Great	N/a	Great		Good	Great	Great		Great	Good			Good	Great
When paused		Great	N/a	Great		Good	Great	Great		Great	Good			Good	Great
Cardiac activity present		No	N/a	Yes		No	No	Minimal		Minimal	No			No	Yes
Aortic valve opening		No	N/a	Yes		No	No	No		No	No			No	Yes
LV function		Absent	N/a	Greatly reduced		Absent	Absent	Minimal		Minimal	Absent			Absent	Mildly reduced
RV function		Absent	N/a	Reduced		Absent	Absent	Absent		Absent	Absent			Absent	Greatly reduced
Pericardial effusion		No	N/a	No		Massive	No	No		No	No			No	No
Complications		None	N/a	None		None	None	None		None	None			None	None
Interference with ALS		No	N/a	No		Yes	No	No		No	No			No	No
Remarks		None	No TEE (power failure)	Pseudo pea		Unexpected pathology (pericardial effusion)	None	None		None	None			None	Severe RV dilatation, thrombolysis

## Discussion

Our study demonstrates the feasibility of TEE to guide treatment of cardiac arrest at OHCA emergency scenes. In experienced hands, TEE allows reversible causes of cardiac arrest to be diagnosed in these situations without adding any adverse effects and without being disruptive to simultaneous activities of resuscitation.

Focused ultrasound examinations are today routinely used by emergency and intensive care physicians both in most hospitals and in prehospital settings^5^. There are, however, limitations to transthoracic electrocardiography in situations of cardiac arrest. Notably in mechanically ventilated patients, its image quality is inferior compared to TEE^9–11^. In addition, the time windows for transthoracic ultrasound are confined to pauses in chest compression (e.g. for rhythm checks). Studies have indicated a doubling of hands-off times, which implies significant interruptions in chest compressions, by transthoracic electrocardiography^7,8^. This drawback was not observed with TEE^16^.

The present study, too, yielded hands-off times that were statistically comparable to a historical control group not involving point-of-care ultrasound. Also, the proportion of cases in which TEE prompted changes in clinical management was considerable. Arguably, these findings of severe right ventricular dilatation (n = 1), pericardial effusion (n = 1), or return of spontaneous circulation (n = 1) might also have been detectable by transthoracic echocardiography. TEE is, however, known from previous studies to offer superior identification of relevant pathologies in critically ill, ventilated patients^9,10^. As additional benefits, chest compressions can be viewed and optimized for effectiveness in real time, and the TEE probe can be left in situ after return of spontaneous circulation for continuous visualization of cardiac function in this precarious phase^14,15,17–22^. Despite these potential benefits, the TEE probe was withdrawn in 3 cases. In one case, the focus was switched to transport priority under CPR. In two cases, the treating physicians did not believe, that keeping the probe in situ would add additional information and therefore should be removed.

We invariably achieved excellent or good image qualities during chest compressions in a study environment that included an extra emergency physician with TEE expertise. An expert was added because the skill to conduct TEE safely and effectively, which does require special training, is not a given amongst prehospital emergency physicians in our city^23^. While this reliance on extra staff might be considered a limitation of our study, the learning curve for TEE in intubated patients has not been found to be overly steep, with 20–50 procedures being required for successful examinations in 80–100% of cases^24,25^.

In-hospital settings have been shown previously to enable safe application of TEE by emergency physicians and intensive care specialists, frequently disclosing valuable information that prompted changes in clinical management^13,26^. Based on what evidence is currently available, we expect that TEE can soon be implemented at OHCA emergency scenes, maybe without a need to dispatch extra staff. We also expect the findings of the present investigation to set a departure point for larger-scale outcome studies.

In summary, this study demonstrates both the feasibility of TEE examinations at OHCA emergency scenes in a vast majority of cases and the ability of findings thus obtained to prompt changes in clinical management decisions. Further studies are needed to address in greater detail questions related to the training of operators, implementation of TEE in routine care, impact on hands-off times, and improvement of patient outcomes.

## Data Availability

The individual de-identified participant datasets generated and analysed during the current study are available from the corresponding author on reasonable request.
